# Externally validated population pharmacokinetics of amikacin and evaluation of dosage regimen based on achieved serum concentrations in neonates

**DOI:** 10.1128/aac.00818-25

**Published:** 2025-07-17

**Authors:** Chuan Poh Lim, Samuel Rocky Candra, Sheng Hsuan Tseng, Ebenezer Priyantha Edison, Mary Grace Sy Tan, Mei Hui Amanda Yong, Yufei Chen, Cheo Lian Yeo

**Affiliations:** 1 Division of Pharmacy, Singapore General Hospital 37581, Singapore, Singapore; 2 Duke-NUS School of Medicine, Singapore, Singapore; 3 Department of Neonatal and Developmental Medicine, Singapore General Hospital 37581, Singapore, Singapore; 4 Yong Loo Lin School of Medicine, National University of Singapore 63751 , Singapore, Singapore; 5 Lee Kong Chian School of Medicine, Nanyang Technological University 54761, Singapore, Singapore; Providence Portland Medical Center, Portland, Oregon, USA

**Keywords:** amikacin, neonates, population pharmacokinetics, route of administration, external validation

## Abstract

Combination antibiotics consisting of beta-lactam and aminoglycoside are commonly utilized in the treatment of neonatal septicaemia. The aims of this study were to (i) develop an externally validated population pharmacokinetic (PK) model, (ii) evaluate the attainment of target peak and trough serum concentrations by the current hospital amikacin dosing protocol and side effects, and (iii) compare intravenous (IV) versus intramuscular (IM) route of amikacin administration, in terms of attaining peak and trough serum concentration targets. Retrospective chart review was carried out over a 5-year period. All neonates who received amikacin with therapeutic drug monitoring performed were included in this study. A one-compartment population PK model was built, and external validation was performed. A total of 181 neonates (534 serum concentrations) were included in the population PK modeling and external validation. There was no apparent systematic bias in the predictions of the model. The external validation performed in the current study found the model to be generally unbiased. Sixty-one percent of the peak and 99% of the trough levels were within the targeted therapeutic ranges of 15–25 and <5 mg/L, respectively. There was no statistical difference in the proportion of trough concentrations that were within therapeutic range for IV as compared to IM, while IM resulted in a higher proportion of trough concentrations within therapeutic range, as well as higher peak concentrations. The current population PK model and external validation study have proven that the PK model built in the current study can be used to conduct reliable population simulations. IM injection can be an alternative route of administration for amikacin in neonates.

## INTRODUCTION

Bacterial infection is common in neonates, especially in preterm neonates due to their immature immune system (1). Aminoglycosides are commonly used in combination with beta-lactam in the treatment of neonatal septicaemia, both early onset and late onset sepsis. The spectrum of activity of aminoglycosides includes coverage for both gram-negative bacilli as well as additive or synergistic activity for gram-positive organisms when used in combination with a beta-lactam. Amikacin, a semisynthetic derivative of kanamycin, was specifically developed to prevent bacterial plasmid-mediated enzyme degradation (2). A major advantage of amikacin is its effectiveness against gram-negative bacteria that are resistant to gentamicin and tobramycin (3–6).

Amikacin exhibits concentration-dependent killing properties as well as post-antibiotic effect (PAE). Target peak concentration of 20–30 mg/L and trough concentration of 2–5 mg/L are quoted as measures of efficacy and safety for amikacin (7). Amikacin dosing regimen used differs among neonatal units, with the majority based on postmenstrual age (PMA) and/or postnatal age (PNA) (8–11). In addition, current evidence recommends the use of aminoglycoside extended dosing interval (dosage interval typically 24 hours in term and 36–48 hours in preterm neonates) as compared to traditional dosing (dosage interval typically 8–12 hours in term and 12–24 hours in preterm neonates), in order to reduce the risk of serum drug concentrations outside the therapeutic range (12). Despite controversies in aminoglycoside-induced nephrotoxicity and ototoxicity in neonates on amikacin, monitoring of serum amikacin concentrations remains recommended. This ensures nil drug accumulation.

At the Department of Neonatal and Developmental Medicine, Singapore General Hospital (SGH), the institutional dosage regimen for amikacin is based solely on PMA, with peak and trough serum levels obtained pre- and post-third dose. These serum levels are used to guide subsequent dosing. Guided by the antibiogram of the organisms in the institution, targets for peak and trough amikacin serum concentration of 15–25 and <5 mg/L, respectively, were recommended to ensure safe and effective therapy for neonates on amikacin therapy.

The primary aim of the study was to develop an externally validated amikacin population pharmacokinetic (PK) model in the neonatal population. The secondary aims of the study were to evaluate the attainment of target peak and trough serum concentrations by the current hospital amikacin dosing protocol and side effects, as well as to compare differences in therapeutic thresholds attained in neonates on intramuscular (IM) versus intravenous (IV) amikacin.

## RESULTS

**Fig 1:**
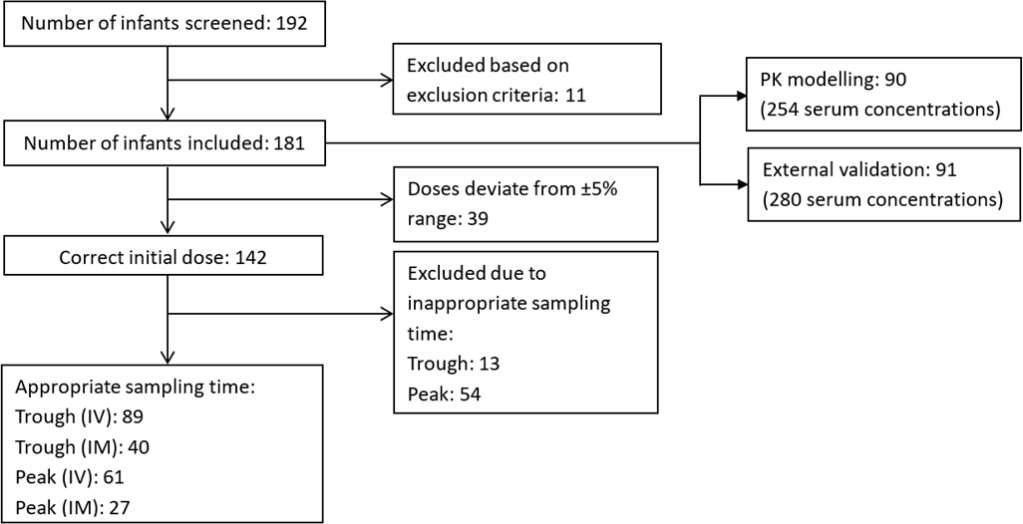
Flow chart of study subjects.

A total of 181 neonates fulfilled the inclusion and exclusion criteria of this study; data from 90 neonates (254 serum concentrations) were utilized for PK modeling, and external validation was performed using data from the remaining 91 neonates (280 serum concentrations). Of the 181 neonates, 142 neonates received the recommended institutional amikacin dosage regimen with serum concentration monitored at appropriate timing (Fig. 1). Patient demographics and characteristics for both PK modeling and external validation were summarized in Table 1.

**TABLE 1:** Demographics and characteristics of study subjects^*a*^

Variables	PK modeling *n* = 90	External validation *n* = 91
Male, *n* (%)	56 (62.2)	50 (54.9)
Race, *n* (%)		
Chinese	56 (62.2)	48 (52.7)
Malay	12 (13.4)	23 (25.3)
Indian	11 (12.2)	14 (15.4)
Others	11 (12.2)	6 (6.6)
GA, weeks		
median (IQR)	29^+0^ (26^+0^,31^+0^)	29^+0^ (26^+0^,32^+0^)
min, max	24^+0^, 41^+0^	23^+0^, 40^+0^
PMA at initiation, weeks		
median (IQR)	45^+6^ (39^+5^,53^+5^)	42^+6^ (36^+6^,51^+3^)
min, max	25^+6^, 113^+6^	27^+2^, 91^+3^
Birth weight, kg		
median (IQR)	1.00 (0.87,1.44)	1.12 (0.84,1.64)
min, max	0.54, 3.77	0.32, 3.48
Weight at initiation, kg		
median (IQR)	1.34 (0.90,1.90)	1.39 (0.88,1.79)
min, max	0.58, 3.77	0.32, 3.47
SGA, n (%)	23 (25.6)	11 (12.1)
IUGR, n (%)	10 (11.1)	9 (9.9)
SCr at initiation, µmol/L		
median (IQR)	43 (29,57)	45 (31,58)
min, max	15, 91	18, 109
Concomitant nephrotoxic agent, n (%)		
Aciclovir	0 (0.0)	0 (0.0)
Ibuprofen	4 (4.4)	3 (3.3)
Indomethacin	0 (0.0)	0 (0.0)
Fluconazole	1 (1.1)	29 (31.9)

^*a*^ GA, gestational age; IQR, interquartile range; IUGR, intrauterine growth restriction; PMA, postmenstrual age; SCr, serum creatinine; SGA, small for gestational age.

### Population PK analysis

A one-compartment model with first-order elimination adequately described the amikacin concentration time course in the study subjects. An exponential error model for between-subject variability (BSV) and a combined proportional-additive error model for residual unexplained variability (RUV) resulted in the largest decrease in the objective function value (OFV; i.e., the best model fit) for the base model.

All covariates tested in univariate analysis were supported by individual Bayesian PK parameter estimates and residual versus covariate plots. The final population model selected after covariate screening and forward addition/backward elimination comprised the following equations:


$$\begin{array}{l} CL\;(L/H)=0.0487(WT/1,000{)}^{0.919}(PMA/30{)}^{1.53}exp[\eta ∼N(0,\sigma^{2}=0.0188)]\\ V(L/kg)=0.497(WT/1,000{)}^{0.851} \end{array}.$$

Standard diagnostic plots, prediction-corrected visual predictive check (pcVPC), and bootstrap results were satisfactory and did not raise any major concerns of model misspecification or imprecision.

### Prediction-based diagnostics for external validation

**Fig 2:**
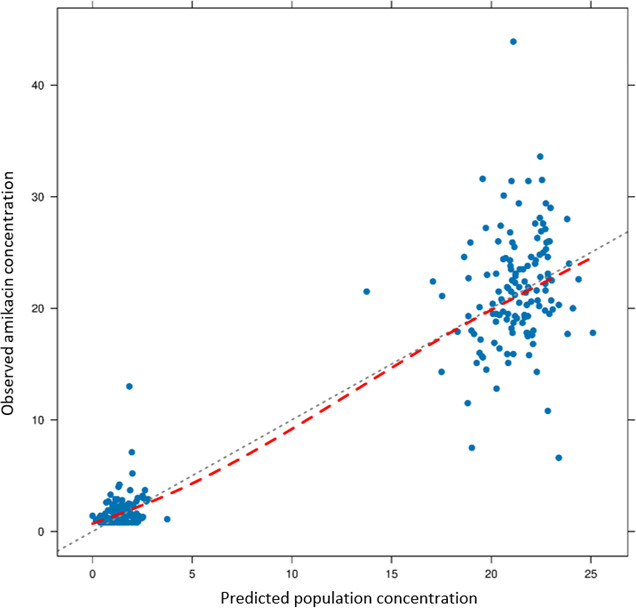
Plot of observed concentration (mg/L) against population predicted concentration (mg/L). The dotted black line represents the line of equity, while the dashed red line represents the trend line.

A diagnostic plot of predicted population concentration (PRED) vs observed amikacin concentration (OBS) (Fig. 2) showed that the data points were generally scattered uniformly around the line of equity. Metrics to quantify predictive performance were as follows:

MPE% = −4.99%

MAPE% = 22.46%

RMSE% = 48.82%

F_20_ = 44.64%

F_30_ = 64.29%

All metrics except for RMSE% passed the predefined criteria for the model to be considered to have satisfactory predictive performance.

### Simulation-based diagnostics for external validation

**Fig 3:**
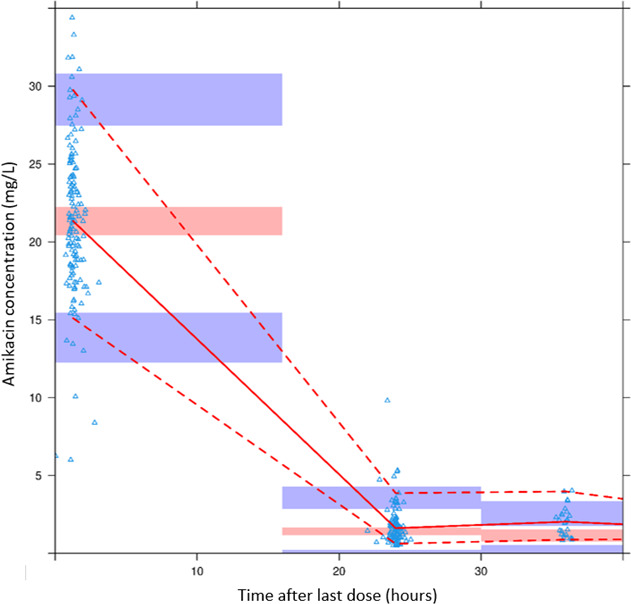
pcVPC for the final population PK model. The solid red line represents the median prediction-corrected observations, and red bands represent the simulation-based 95% CI for the median. Dashed red lines represent the prediction-corrected observed 5th and 95th percentiles, and purple bands represent the simulation-based 95% CIs for the corresponding model-predicted percentiles. The prediction-corrected observations are represented by blue triangles.

**Fig 4:**
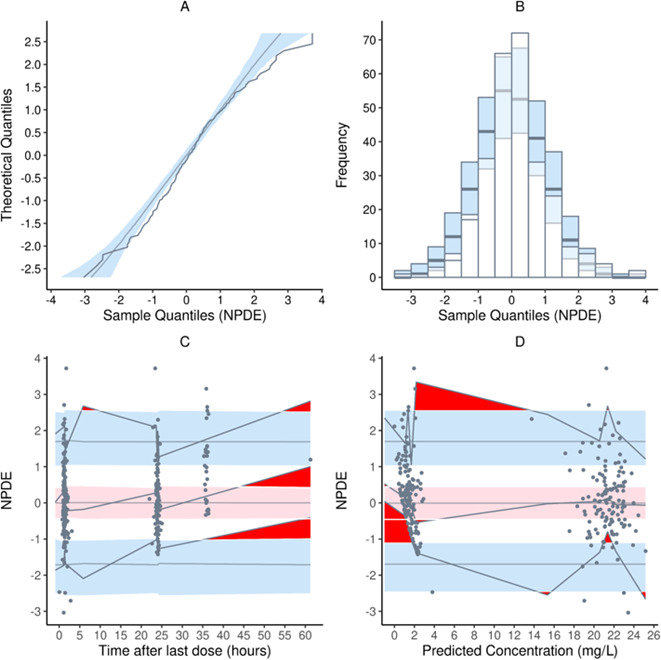
NPDE plots of the model: (**A**) quantile-quantile plot of the theoretical distribution (blue band) against the distribution of the NPDE; (**B**) histogram of the theoretical distribution (blue bars) overlaid on the distribution of the NPDE; (**C**) NPDE vs time after last dose; and (**D**) NPDE vs predicted concentrations. In plots **C** and **D**, the middle solid grey line represents the median NPDE of the observations, and the red band represents the simulation-based 95% CI for the median. Top and bottom black lines represent the NPDEs of the observed fifth and 95th percentiles, while blue bands represent the simulation-based 95% CIs for the corresponding model-predicted percentiles. The NPDEs of the observations are represented by black dots.

In the plot generated after pcVPC (Fig. 3), most of the prediction-corrected observed concentrations were generally predicted without bias by the model. The normalized prediction distribution error (NPDE) distribution of the model did not fully pass the test for normality due to Shapiro-Wilks test and the global test having *P* values of <0.05 (Table 2; Fig. 4).

**TABLE 2:** Results of NPDE analysis

Mean	Variance	Wilcoxon signed rank test for means	Fisher’s variance test	Shapiro-Wilks test for normality	Global test^*a*^
		*P* value	*P* value	*P* value	*P* value
0.133	0.987	0.214	1	<0.001	<0.01

^*a*^ The global test *P* value is reported as the minimum of the previous three *P* values multiplied by 3.

**Fig 5:**
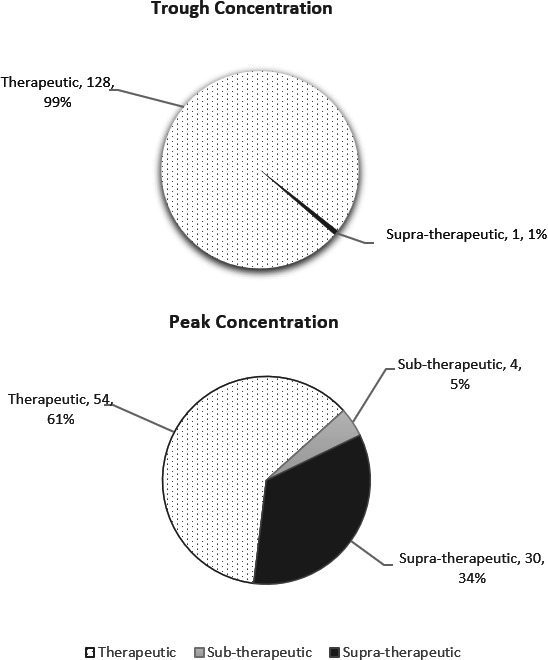
Amikacin serum concentrations.

Of the 142 neonates who received the recommended institutional amikacin dosage regimen with serum concentration monitored at appropriate timing, there were 129 trough concentrations and 88 peak concentrations performed at the appropriate timing and included in the comparison between IV and IM routes of administration. Of these serum concentrations, 61% of the peak and 99% of the trough levels were within the targeted therapeutic ranges of 15–25 and <5 mg/L, respectively (Fig. 5).

In terms of route of administration, there was no difference in the proportion of trough concentrations that were within therapeutic range for IV as compared to IM, while IM resulted in a higher proportion of trough concentrations within therapeutic range, as well as higher peak concentrations (Table 3). Thirty-seven (26.1%) neonates required dosage adjustment for amikacin, and seven (4.9%) neonates required a change in antibiotic therapy: two of whom had antibiotic escalated to a broader spectrum antibiotic, four changed to culture-directed antibiotic, and one changed to an alternative antibiotic due to error in amikacin administration.

**TABLE 3:** Comparison of trough and peak serum concentration levels of subjects on IV vs IM amikacin

Serum concentration	IV	IM	P
Trough, mg/L (mean ± SD)	1.62 ± 0.73	1.65 ± 0.97	0.843^*a*^
Within therapeutic range, %	98.9	100.0	1.000^*b*^
Peak, mg/L (mean ± SD)	22.23 ± 4.89	24.46 ± 5.15	0.055^*a*^
Within therapeutic range, %	65.6	48.1	0.123^*b*^

^*a*^ Independent sample *t*-test.

^*b*^ χ^2^ test or Fisher’s exact test where appropriate.

Eleven (7.7%) neonates failed the newborn hearing screening using the two-staged otoacoustic emissions (OAE) and automated auditory brainstem response (AABR) protocols and were referred for audiological evaluation. Two of them had bilateral moderate sensorineural hearing loss requiring cochlear implants. This included one neonate with congenital cytomegalovirus infection and a preterm neonate with perinatal stroke secondary to protein C deficiency who had bilateral severe sensorineural hearing loss. Excluding one neonate, all were exposed to gentamicin in addition to amikacin for a prior episode of sepsis during the inpatient stay. Serum amikacin drug levels were normal in nine neonates; two had high peak concentrations of 27.2 and 25.3 mg/L, and their serum gentamicin levels were normal. None of the neonates with significant hearing impairment or hearing impairment requiring further management had supra-therapeutic amikacin levels. None of the neonates on amikacin therapy had renal impairment.

## DISCUSSION

In the present study, we built a one-compartment population PK model for amikacin using data from 90 neonates. Either one- or two-compartment models were used in the previous PK studies to describe amikacin pharmacokinetics in the pediatric population. Our study found a better fit of data with the one-compartment model produced. The results from our study confirmed previous findings that PMA and weight (WT) are the main factors affecting amikacin pharmacokinetics in neonates (13–17). An external validation study by Matcha et al. (18) compared 12 published amikacin population PK models. In this study, the PK model with the best predictive performance reported body WT and creatinine clearance (CL) as the significant covariates impacting amikacin pharmacokinetics (19). Our study did not include creatinine CL but PMA, as neonatal renal maturation correlates with their age (20).

To ensure the generalizability of our newly developed population PK model, an external validation was performed, using another group of 91 neonates who were not involved in the development of the PK model. The US Food and Drug Administration (FDA) described external validation of a population PK model as the gold-standard evaluation method for a developed model. However, it is performed for <10% of published population PK models, limiting the clinical utility of these models (21). To the best of our knowledge, the current amikacin PK model involved the largest number of neonates, with external validation performed.

In external validation, bias and precision are the main parameters of interest. If either one is not satisfactory, the validity of results derived from simulations or Bayesian predictions from the model would be deemed unreliable. Taking all diagnostics into consideration, the external validation of our population PK model was deemed to be satisfied despite not fulfilling all the passing criteria. In addition, the PK model attempts to account for unknown covariates that add to variability, by characterizing them in the form of a log-normal distribution with a known variance. Unfortunately, one disadvantage of modeling and simulation is that only theoretical results are derived and need to be tested in the clinical setting on actual subjects. Besides, these results are affected by the underlying bias and precision of the PK model, hence the need for external validation.

As amikacin exhibits concentration-dependent kill, the antimicrobial effect can be optimized through administration of a larger dose to achieve higher serum concentration. Once-daily dosing of amikacin has been found to be more efficacious as well as a better safety profile (12). However, the commonly used drug reference in neonates, NeoFax, recommends amikacin frequency to be prolonged to 36 or 48 hours for neonates with PMA <35 weeks in their first week of life and first 28 days in those with PMA <30 weeks, with a slightly higher dosage recommendation for different age group (7).

PAE allows antibiotic doses to be administered less frequently. The PAE of amikacin on *Staphylococcus aureus*, *Escherichia coli*, and *Pseudomonas aeruginosa* was studied by Renneberg and Walder (22), both *in vitro* and *in vivo*. In the *in vivo* tests, the mean times above minimal inhibitory concentration (MIC) was 0.8–1.0 hour. The mean PAE durations for all three pathogens range from 1.4 to 3.8 hours. When the same pathogens were exposed to amikacin at a concentration of 10 times above the MIC for 2 hours *in vitro*, the mean amikacin PAE durations were found to be 1.5–2.2 hours. It remains unknown whether amikacin serum concentration drops to a sub-therapeutic level if the dosing frequency is extended beyond 24 hours, thereby affecting its efficacy. Future studies should focus on determining the most appropriate dosage regimen through Monte Carlo simulation using an externally validated PK model.

Findings of the current study showed that 61% of peak and 99% of trough concentrations were within the therapeutic range. IM amikacin administration was found to be similar to the IV route, in terms of the serum concentration achieved in this study. Our finding is in alignment with that of a previous amikacin PK study in neonates (23). A total of 11 neonates who received amikacin failed the inpatient newborn hearing screening done using OAE and/or AABR. Swelling of nerve terminals within 15–18 hours post-exposure, as well as death of inner ear hair cells 1–2 days following exposure, was found to be associated with aminoglycoside-induced ototoxicity (24, 25). Previous studies suggested that ototoxicity is more likely when amikacin trough level is ≥10 µg/mL (26–28). Another study found that mitochondrial DNA mutations were linked to aminoglycosides sensitivity and associated with hearing loss in the absence of exposure to aminoglycosides (29).

None of the neonates on amikacin therapy had renal impairment in this study. More than 90% of the aminoglycosides dose is eliminated through the kidneys (30, 31). Aminoglycoside-induced nephrotoxicity correlates with the total dose and duration of treatment. The condition is reversible due to the capacity of proximal tubular cells to regenerate (32, 33). Neonates have a lower rate of nephro- and ototoxicity as compared to adults, in view of maturation-related low expression of ligands (megalin and cubulin) that facilitate the uptake of aminoglycosides at the surface of renal tubular and cochlear hair cells (34).

Our study was not without limitations. First, the retrospective nature of this study could compromise the quality of data. There was a huge reliance on the documentation of blood sampling and dose administration time by the nurses. Second, missing data for serum creatinine (sCr) and body WT was imputed using the last observation carried forward technique. However, most of the neonates had their renal panel and body WT checked at least once during the course of amikacin treatment. Based on the results, there was a good correlation between body WT and volume of distribution (V), which matches our expectation.

In conclusion, the current population PK model and external validation study have proven that the PK model built in the current study can be used to conduct reliable population simulations. IM injection can be an alternative route of administration for amikacin in neonates. With the externally validated amikacin PK model, further research can be carried out to develop optimal amikacin dosing regimens in neonates.

## MATERIALS AND METHODS

This study utilized data set from a previous study (35). All neonates managed in the Department of Neonatal and Developmental Medicine, SGH, over the period of November 2012 and October 2017 who received amikacin were included in this retrospective chart review. Approval to conduct this study was granted by the SingHealth Centralised Institutional Review Board (reference 2018/2546) with waiver of parental consent. Amikacin doses were administered via IV infusion over a nominal duration of 30 minutes or IM injection and dose based on the neonate’s PMA: 11 mg/kg every 36 hours and 11 mg/kg every 24 hours in neonates <29 weeks and ≥29 weeks, respectively. Therapeutic drug monitoring was performed at the third dose of the dosing regimen with trough concentrations taken within 30 minutes preceding a dose, and peak concentrations were taken 60 minutes after IM injection, or 30 minutes after the end of an IV infusion. Random concentrations were also considered if the timings of blood sampling were documented.

Data for all eligible neonates were collected from electronic medical records using Redcap and included demographics, sCr, the complete amikacin dosing and concentration history, indications for amikacin use, routine hearing test results, and information regarding concomitant nephrotoxic agents. The routine hearing test was carried out as part of the universal newborn hearing screening program for all infants prior to hospital discharge. Neonates with ambiguous amikacin dosing and serum concentrations, congenital kidney disease, major congenital heart disease (diagnosis other than ventricular septal defect, atrial septal defect, or patent ductus arteriosus), acute kidney injury, or unstable renal function and those on extracorporeal membrane oxygenation during amikacin therapy were excluded. The WT of the neonate at amikacin initiation was recorded. The last measured WT would be carried forward if no measurement was done on the day of amikacin therapy initiation. sCR levels around the time (±48 hours) of all amikacin concentration measurements were recorded and updated throughout the amikacin course. Where the sCr readings within ±48 hours of assay of amikacin concentrations were unavailable, the closest available sCr reading was imputed.

We defined the correct amikacin dose as ±5% of the calculated dose based on the WT at amikacin therapy initiation. Appropriate trough concentration sampling time was defined as ≤30 minutes preceding a dose, while appropriate peak sampling time was defined as 60–90 minutes after an IM injection or initiation of an IV infusion. Both peak and trough concentrations were deemed appropriate when done on or after the third dose of amikacin. The serum concentration targets were 15–25 mg/L for peak and <5 mg/L for trough concentration.

In the analysis, categorical data were reported as number (percentages). Normally distributed data were reported as mean ± standard deviation, while nonnormally distributed data were reported as median (interquartile range). For comparison between IM versus IV route of administration, χ^2^ test or Fisher’s exact test was used for categorical data, while continuous data were analyzed using independent sample *t*-test. A *P* value of <0.05 was considered statistically significant.

### Population PK analysis

Population PK analysis was performed using nonlinear mixed effects modeling software NONMEM (NONMEM 7.5; Icon Development Solutions, Ellicott City, MD, USA). As most of the amikacin concentrations were measured after the distribution phase, a one-compartment model was used as the structural model. The first-order conditional estimation method with interaction was implemented throughout the model building and evaluation process. RUV was evaluated using additive, proportional, and combined proportional-additive error models, while BSV was evaluated based on V (expressed as liters/kg) and CL (expressed as liters/hour) using an exponential error model.

Multiple covariates related to maturation (age), size (WT), and renal function were evaluated for their impact on amikacin PK. Normalization of all continuous variables was carried out during the modeling process. The effect of WT (expressed in grams) on the V and CL was explored using allometric and power models. Covariates related to age included gestational age (GA [weeks]), PMA (weeks), and PNA (days), and their effect on CL was explored using a sigmoid maximum effect maturation function (for PMA only) and power models. The only covariate related to renal function, sCr, was assessed for its effect on CL using a power model. Potential final models with incorporated covariates were evaluated via stepwise forward addition/backward elimination to ascertain the statistical significance of each covariate. In the forward addition step, a covariate would be retained after addition to the model if the decrease in OFV was >3.84 [$X^{2}$ distribution, *P* < 0.05, degree of freedom (df) = 1]. A more stringent criterion was used for the backward elimination step, where a covariate would be retained if the increase in OFV after removal of the covariate was >10.83 ($X^{2}$ distribution, *P* < 0.001, df = 1). The final model was selected based on physiological plausibility of population CL and V estimates, acceptable standard errors, and good visual representation of standard diagnostic plots.

### Model evaluation

Nonparametric bootstrap analysis and a pcVPC were conducted to evaluate the accuracy and stability of the final model using the NONMEM support software Perl-speaks-NONMEM (v5.3.0). For bootstrap analysis, 2,000 bootstrap data sets were generated from the original data set by repeated sampling with replacement. Next, the final model was used to generate parameter estimates for each resampled data set. The medians and 95% confidence intervals (CIs) of bootstrap parameter estimates were compared to their respective values obtained from the original data set. As for pcVPC, 500 virtual data sets were simulated using the final model. The 2.5th, 50th, and 97.5th percentiles of the simulated concentrations were overlaid on the observed concentrations versus time profile using R.

### External validation

The population PK model built in this study was externally validated using prediction-based and simulation-based diagnostics.

#### Prediction-based diagnostics

To evaluate the predictive performance of the model, predicted population concentrations (PRED) were compared to corresponding observed amikacin concentrations (OBS). The median prediction error percentage (MPE%) was calculated to assess bias, while the median absolute prediction error percentage (MAPE%) and the root mean squared error percentage (RMSE%) were calculated to assess precision. F_20_ and F_30_ were also calculated as combination indices of both bias and precision that indicate the percentage of the prediction error that was within the range ±20% and ±30%, respectively. MPE%, MAPE%, and RMSE% were calculated as follows:


$$\begin{array}{c} \text{ Prediction error }(PE)=\frac{PRED-OBS}{OBS}\times 100\\ MPE\%=\text{ Median of PE }\\ MAPE\%=\text{ Median of }|PE|\\ RMSE\%=\sqrt{\frac{1}{N}\sum_{i=1}^{N}(PE{)}_{i}^{2}} \end{array}$$

When MPE% ≤ ±15%, MAPE% ≤ 30%, RMSE <15%, F_20_ >35%, and F_30_ >50% were achieved, the model was considered to have satisfactory predictive performance in terms of bias and precision (36, 37).

#### Simulation-based diagnostics

A pcVPC (38) was performed, where 500 simulated data sets were generated using the model to be evaluated. The pcVPC was executed using the Perl speak NONMEM toolkit (PsN, version 5.3.0). Processing of the pcVPC results and plot generation were performed using the R package “xpose4” (https://cran.r-project.org/web/packages/xpose4/index.html) in the R software (https://www.rproject.org) and R Studio (https://posit.co/download/rstudio-desktop/).

NPDE analysis was performed using the R package “npde” (https://cran.r-project.org/web/packages/npde/index.html) (39). The model to be evaluated was used to generate 5,000 simulated data sets for the NPDE analysis via the NONMEM control stream. The normality of the NPDE was tested with Wilcoxon’s signed rank test for means, Fisher’s variance test, the Shapiro-Wilks test for normality, and a global test reported as the smallest of the three *P* values multiplied by 3. Quantile-quantile (Q-Q) plots, histograms of NPDE, and scatter plots of NPDE versus time after last dose and NPDE versus predicted concentrations were also generated by the NPDE package in R. When the *P* value of the global test was >0.01, then the NPDE of the model was considered to follow a normal distribution and adequately describe the external validation data set (40).
